# Radical Prostatectomy: Evolution of Surgical Techniques from Laparoscopy to Robotics

**DOI:** 10.3390/jcm14103444

**Published:** 2025-05-15

**Authors:** Tomasz Syryło, Tomasz Ząbkowski, Tomasz Waldemar Kamiński, Ryszard Skiba, Hubert Andrzej Krzepkowski

**Affiliations:** 1 Department of General, Oncological and Functional Urology, Military Institute of Medicine–National Research Institute, 04-141 Warsaw, Poland; 2 VERSITI Blood Research Institute, 8727 W Watertown Plank Road, Wauwatosa, WI 53226, USA

**Keywords:** duration of surgery, laparoscopy, prostate cancer, robotic surgery, urinary incontinence

## Abstract

**Background/Objectives**: Radical prostatectomy is a standard treatment for localized prostate cancer. We aimed to compare perioperative outcomes and functional results between laparoscopic radical prostatectomy (LRP) and robot-assisted radical prostatectomy (RARP). **Methods**: A retrospective analysis was conducted on 120 patients who underwent LRP (n = 60) or RARP (n = 60). Perioperative parameters, including operative time, hospitalization duration, blood transfusion rate, wound healing complications, urinary catheterization duration, urinary tract infections (UTIs), and urinary incontinence, were assessed. Statistical analyses included Student’s t-, Mann–Whitney U, and χ^2^ tests. **Results**: RARP was associated with significantly shorter operative time, compared with LRP (147.25 vs. 188.30 min, *p* < 0.0001). No significant differences were observed in hospitalization duration, transfusion rates, or overall complication rates. However, impaired wound healing was less frequent in the RARP group, with a 10% lower incidence, compared with the LRP group (*p* = 0.0946). Similarly, UTIs occurred less often in the RARP group (six vs. one cases; *p* = 0.0544). Urinary incontinence was significantly less frequent following RARP, with its incidence being more than twice as low, compared with the LRP group (*p* = 0.0032). Additionally, the RARP group had significantly lower International Prostate Symptom Scores, with a mean difference of 7.83 points, indicating improved urinary function. No significant differences were found in sexual function (IIEF-5 scores). **Conclusions**: RARP offers advantages over LRP, including reduced operative time, lower rates of wound healing complications, decreased incidence of urinary incontinence, and improved postoperative urinary function. Further studies with larger cohorts are warranted to confirm these findings and assess long-term functional and oncological outcomes.

## 1. Introduction

Prostate cancer is a critical health challenge worldwide. It is a common cancer and a major cause of cancer mortality [1]. Similarly, it remains a commonly diagnosed cancer worldwide, with 2021 data indicating that it is the third most diagnosed cancer and the eighth leading cause of cancer deaths in the male population [2]. Morbidity and mortality rates have recently decreased or remained at a similar level [3]. Additionally, in Poland, prostate cancer is the most prevalent cancer in men and the second leading cause of death [4]. Radical prostatectomy, either as a standalone treatment or in combination with chemotherapy and radiotherapy, constitutes a comprehensive therapeutic approach to prostate cancer, tailored to the stage and aggressiveness of the tumor to optimize treatment outcomes [5]. It is essential to select an appropriate therapeutic approach that maximizes patient benefit while minimizing adverse effects. Radical prostatectomy can be performed using open, laparoscopic, or robotic methods. These therapeutic methods are characterized by distinct postoperative outcomes, which may vary in terms of efficacy, complication rates, and overall impact on patient recovery and quality of life. Minimally invasive procedures have dominated classical surgical techniques [6]. Robotic surgery holds significant promise for improving surgical outcomes by offering enhanced visualization and greater precision during procedures [7]. This advanced technology facilitates superior delineation of fine anatomical structures, thereby enabling more accurate and meticulous surgical manipulation [8]. Concurrently, advances in genetic research, such as the identification of Y chromosome loss, have provided valuable insights into prostate cancer progression. When integrated with modern surgical techniques, including robotic-assisted and laparoscopic prostatectomy, these genetic markers may support the development of more personalized treatment strategies. By combining genetic profiling with state-of-the-art surgical approaches, radical prostatectomy could be tailored to individual patients, thereby optimizing outcomes while minimizing procedural risks [9]. Observations from scientific studies comparing robotic and laparoscopic surgeries differ, possibly owing to differences in the skills of surgical teams and the relatively recent emergence of robots in operating rooms [10]. In this study, we sought to conduct a comprehensive comparative analysis of robotic and laparoscopic prostatectomy techniques, aiming to determine the potential superiority of one of the examined surgical approaches based on clinical outcomes and postoperative recovery parameters.

## 2. Materials and Methods

This study is a retrospective analysis of patients who underwent radical prostatectomy at the Department of General, Oncological and Functional Urology at the Military Institute of Medicine–National Research Institute in Warsaw. The study involved a group of 120 patients aged 53–86 years, who were qualified for radical prostatectomy. Sixty patients were eligible for each of the radical laparoscopic prostatectomy and da Vinci robot-assisted radical prostatectomy. Data were collected from the hospital information system after the patients had been hospitalized and via telecommunications. We aimed to identify the differences between the robotic and laparoscopic surgical methods.

This study focused on individuals who underwent prostatectomy between 2021 and 2024. Patients were not recruited sequentially; instead, a random selection method was employed wherein medical records were randomly chosen from both surgical groups. This approach was adopted to minimize selection bias, enhance the generalizability of the findings, improve sample representativeness, and control for potential confounding variables, such as age and baseline health status. In surgical procedures performed utilizing the da Vinci robotic system, the transperitoneal approach was employed as the primary method of access. In contrast, laparoscopic procedures were conducted using the extraperitoneal approach.

The comprehensive data analysis encompassed a wide range of clinical and demographic parameters, including patient age, total duration of hospitalization, length of the surgical procedure, postoperative wound healing process, length of stay in the postoperative ward, necessity for reoperation, total volume of transfused erythrocyte mass, mortality rates, assessment of lower urinary tract symptoms based on the International Prostate Symptom Score (IPSS), evaluation of erectile function using the International Index of Erectile Function-5 (IIEF-5), incidence of urinary tract infections, prevalence of urinary incontinence, occurrence of postoperative complications, as well as the total duration of urinary catheterization after hospital discharge.

Of the 120 patients, 33 and 34 responded to the telephone survey, and 20 and 19 participated in the assessment of sexual function in the laparoscopic and robotic groups, respectively. The telephone survey was conducted from the hospital department, with all respondents voluntarily consenting to participate in the study after being informed about its objectives and assured of their anonymity. The survey was administered between the third and fourth months postoperatively. The survey collected the following data: assessments based on of the IIEF-5 and the IPSS. Additionally, the questionnaire addressed urinary tract infections, with patients being asked about positive urine culture results and the presence of infection-related symptoms. Any form of urinary incontinence reported by the patient, regardless of its type or severity, was classified as an adverse event. The total duration of catheterization was recorded starting from the day of hospital discharge.

Patients undergoing robot-assisted radical prostatectomy (RARP) were deemed eligible for surgery based on criteria established by the National Health Fund. Inclusion was limited to individuals diagnosed with prostate cancer exhibiting a Gleason score of 6–10 (ISUP Grade Group 1–5) points. Eligible patients presented with either localized disease (clinical stage cT1–T2 N0 M0) or locally advanced disease (cT3a–b N0–1 M0), with no evidence of distant metastases (M0), as confirmed by negative bone scintigraphy or whole-body magnetic resonance imaging. Additionally, all patients demonstrated preserved erectile function, defined as an IIEF-5 score >21 points. Patients initially qualified for robotic surgery but found to have extensive intra-abdominal adhesions or a history of prior abdominal surgeries were instead treated with laparoscopic prostatectomy.

Patients undergoing laparoscopic radical prostatectomy (LRP) were qualified based on clinical criteria consistent with localized (cT1–T2) or locally advanced (cT3a) prostate cancer, with no evidence of distant metastasis (M0). Lymph node involvement was either absent (N0) or suspected (N1), as determined by preoperative imaging. The exclusion criteria for the procedure included patient refusal, anesthesiological disqualification, and the presence of disseminated malignancy.

Normally distributed data were expressed as means ± standard errors of the mean, whereas non-Gaussian data were reported as medians (full range). The normality of the distribution was assessed using the Shapiro–Wilk W test. Group differences between the patients’ groups were evaluated using the Student’s *t*-test or the nonparametric Mann–Whitney test, based on the data distribution. Categorical variables were analyzed using the χ^2^ test. Correlations were assessed using Spearman’s Rank correlation analysis. Statistical significance was set at *p* < 0.05 (two-tailed). A *p*-value < 0.1 was considered on the borderline of significance. All computations were performed using GraphPad Prism 10 (GraphPad Software, La Jolla, CA, USA). The study was conducted in accordance with the Declaration of Helsinki and approved by the Institutional Review Board of Military Institute of Medicine–National Research Institute in Warsaw (protocol code 8/25 from 19 February 2025) for studies involving humans.

## 3. Results

The study group comprised two subgroups of 60 people each, all of whom underwent a radical prostatectomy. The first group underwent laparoscopic surgery, whereas the second group was operated on using the da Vinci robot.

**Figure 1:**
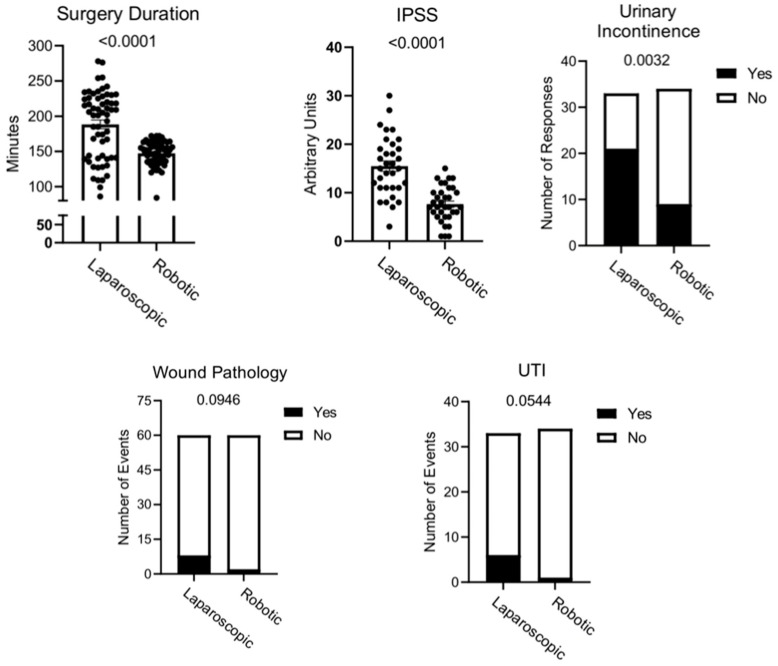
Graphical representation of the key findings of the study. All graphs were generated using GraphPad Prism 10 (GraphPad Software, La Jolla, CA, USA).

Table 1 clearly demonstrates the correlation between the LRP and RARP groups. The research included individuals aged 53–86 years. The median age of patients assigned to the laparoscopic surgery category was established at 70 years, whereas the median age of those undergoing robot-assisted procedures was calculated at 69 years. A comparative statistical assessment of age distribution across the two examined groups did not yield any significant differences, thereby indicating a similar age structure within both populations (*p* = 0.0981). Furthermore, the median duration of hospitalization (*p* = 0.3637), as well as the length of stay in the postoperative care unit (*p* = 0.8155), remained identical between the two groups, measured at 5 days and 1 day, respectively. No significant intergroup difference was detected in the overall hospitalization period, suggesting a comparable postoperative convalescence trajectory in terms of inpatient medical supervision. In both groups there were no instances of mortality during the hospitalization period. Surgical interventions utilizing robotic assistance were conducted in a considerably shorter timeframe, with the operating surgeon requiring, on average, 41 min less to effectively execute the radical prostatectomy procedure compared with the laparoscopic technique (*p* < 0.0001). Additionally, a reduced prevalence of postoperative wound-healing complications was documented among patients subjected to robotic surgery relative to those who underwent laparoscopic interventions. Such complications were recorded in two cases in the robotic-assisted surgery group and eight cases in the laparoscopic surgery group (*p* = 0.0946). The necessity for secondary surgical intervention remained at a comparable rate across both groups, with the robotic-assisted surgery group exhibiting just one fewer instance of reoperation (*p* > 0.9999). Moreover, no discernible differences were observed regarding the requirement for erythrocyte mass transfusion, as both groups recorded a single occurrence necessitating transfusion (*p* > 0.9999). The disparity in the incidence of postoperative complications between the two examined groups was not significant. Within the robot-assisted surgery group, the total count of reported postoperative complications was marginally lower, with a decrease of only two cases relative to the laparoscopic surgery group. This minor numerical reduction does not suggest a meaningful clinical divergence in postoperative outcomes between the two surgical methodologies (*p* = 0.8140). The IPSS assessment showed that patients who underwent robotic surgery reported better well-being and fewer urinary symptoms (*p* < 0.0001). On average, patients in the laparoscopic group rated their symptoms at 15.48/41, whereas patients in the robotic group rated them at 7.65/41 (*p* < 0.0001). Despite the pronounced disparity observed in the IPSS, no significant differences were detected in the evaluation of sexual function across the two analyzed groups. The median score for both groups was recorded as 0, whereas the mean values for patients undergoing robot-assisted and laparoscopic surgery were calculated at 3.21 and 4.95, respectively, demonstrating a slight advantage in favor of the laparoscopic approach based on the IIEF-5 scale (*p* = 0.4828). Concerning the duration of urinary catheterization, no statistically meaningful difference was observed between the two surgical techniques, as the median catheterization period remained consistently at 1 week in both cases (*p* = 0.1521). However, a significant difference was identified in the prevalence of urinary incontinence, with 21 individuals from the laparoscopic surgery group reporting symptoms of this postoperative complication, whereas only nine patients who underwent the robot-assisted procedure reported the symptoms (*p* = 0.0032). An additional noteworthy finding pertains to the incidence of urinary tract infections. Among patients subjected to LRP, six individuals reported experiencing a urinary tract infection, whereas, in the group undergoing robot-assisted surgery, only a single patient reported a urinary tract infection (*p* = 0.0544). A graphical summary of the results is presented in Figure 1.

Table 2 provides a detailed overview of the correlations between the examined parameters in the laparoscopic group. A significant reduction in the number of adverse symptoms, as assessed using by the IPSS, was associated with improved sexual function (*p* < 0.0001). The following correlations merit further consideration and in-depth analysis. A potential relationship was observed between patient age and the duration of the surgical procedure, with older individuals exhibiting a prolonged operative time (*p* = 0.062). Additionally, an extended period of urinary catheterization was correlated with an increased incidence of urinary tract infections, suggesting that catheterization duration may serve as a contributing factor to postoperative infectious complications (*p* = 0.042). Furthermore, a greater number of adverse urinary tract symptoms following surgery was linked to a higher frequency of urinary tract infections, highlighting a potential interdependence between postoperative lower urinary tract dysfunction and infection risk (*p* = 0.011).

Within the group of patients who underwent robot-assisted surgical intervention, a noteworthy correlation emerged that warrants further investigation. Specifically, as shown in Table 3, a prolonged duration of hospitalization appeared to be associated with a potentially increased number of adverse symptoms as evaluated using the IPSS (*p* = 0.078).

## 4. Discussion

The analysis of procedures performed at our center showed the advantage of robotic radical prostatectomy over the laparoscopic method. The comparative analysis of laparoscopic and robot-assisted radical prostatectomy presented in this study yielded several clinically relevant insights that can inform both surgical decision-making and patient counseling. A positive correlation was observed between increasing patient age and longer operative times—a consideration that may be particularly pertinent when selecting an appropriate surgical approach for older patients. Given its association with shorter operative duration, robot-assisted surgery may be preferable in this population. Moreover, the lower incidence of postoperative urinary tract infections in the robot-assisted group supports its use, particularly in patients at higher baseline risk for infection. Functional advantages associated with the robotic approach may also contribute to faster recovery and enhanced postoperative quality of life. Notably, patients undergoing robot-assisted procedures experienced significantly less urinary incontinence and reported fewer lower urinary tract symptoms, as evidenced by improved IPSS.

Despite reports of shorter hospitalizations and stays in the postoperative ward, we observed no similar trends among our patients, whose lengths of stay were identical [11,12]. The duration of the surgical procedure remains deliberated. The duration of radical prostatectomy varies depending on the center [13,14,15]. We have shown a significant difference in favor of the robotic method, with the average duration of the robotic procedure being 147.25 min, whereas that of the laparoscopic procedure was 188.30 min. We assessed the occurrence of pathologies in postoperative wound healing. The postoperative complications evaluated included prolonged wound bleeding, hematoma, surgical site infection, abscess formation, keloid development, and wound dehiscence. Adverse reactions occurred significantly more frequently in the laparoscopic group (13.3%) than in the robotic group (3.3%). The literature is inconsistent regarding the incidence of infectious complications [16,17]. Considering the total number of postoperative complications, the robotic method is superior to the open and laparoscopic methods [18]. The numbers of postoperative complications during the hospital stay and those of required reoperations did not differ among our patients. The overall mortality rate for prostatectomy surgery was 0.13%. Furthermore, the 8-year mortality rate was lower in the robotic group than in the laparoscopic group [19,20]. No deaths were reported in the two groups. Blood loss is likely lower, and blood components require transfusion less frequently during robotic surgery [21,22]. In contrast to this common hypothesis, we observed no differences between our patient groups. Patients who underwent robotic surgery reported better postoperative effects in the form of improved well-being and control of urinary symptoms [23,24,25]. The data collected during the telephone survey showed a significantly better control of urinary symptoms and improved well-being among patients who underwent robotic prostatectomy. The assessment was conducted using the IPSS, where the patients who underwent robotic surgery and LRP scored an average of 7 and 15 points, respectively. Despite reports of better potency among patients from the robotic group, the two groups showed no differences in this study. A study of 140 patients showed that 10% of patients present with symptoms of a urinary tract infection at the time of hospital discharge [26]. Within three years after discharge, six patients in the laparoscopic group experienced a urinary tract infection, compared with only one patient in the robotic group. Patients who undergo robotic surgery are less likely to experience urinary incontinence than are those treated with LRP, particularly in the early period [27,28]. We observed a significant difference in the incidence of urinary incontinence among our patients in favor of patients from the robotic group. Within 3 years postoperatively, urinary incontinence was observed in 26.46% of patients in the robotic group, compared with 63.63% in the laparoscopic group. The durations of catheterization after discharge from the hospital did not differ between the two techniques. Similarly, some of the available publications reported no significant differences between the two surgical techniques [29,30,31,32].

The learning curve in RARP reflects the progressive improvement in surgical outcomes as the operating surgeon accrues experience. Initially, limited familiarity with the robotic system is associated with longer operative times and higher complication rates. However, after approximately 50–60 procedures, surgeons typically achieve more consistent and stable outcomes. The learning process encompasses not only technical proficiency but also non-technical competencies, such as intraoperative team coordination. With increased experience, improvements have been observed in operative efficiency, reduced intraoperative blood loss, and decreased postoperative complication rates. Surgeons who perform the procedure frequently (i.e., high-volume surgeons) tend to achieve superior outcomes due to the cumulative benefits of repeated practice and skill refinement [33,34].

Surgical margin status serves as a critical indicator of oncologic efficacy in nerve-sparing radical prostatectomy. The adoption of robotic technology offers advantages, such as enhanced visualization and instrument precision, which may facilitate more accurate dissection and a reduced risk of positive surgical margins. This is especially relevant in procedures aimed at preserving neurovascular structures, where achieving a balance between oncological control and functional preservation is paramount [35].

Although robotic systems differ in their technical specifications and design, recent clinical experiences with newer platforms, such as the Hugo RAS system, highlight the advantages of RARP in terms of intraoperative performance and early postoperative recovery. Reported outcomes include reduced operative duration, minimal blood loss, low complication rates, and improved early functional results, particularly in relation to urinary continence. These benefits are attributed to the enhanced surgical precision, superior visualization, and increased dexterity inherent in robotic-assisted procedures. The reproducibility of these outcomes across various robotic platforms underscores the growing clinical preference for robotic techniques, particularly when early recovery and continence preservation are prioritized in surgical planning [36]. Moreover, robot-assisted radical prostatectomy offers distinct advantages over conventional laparoscopic techniques, particularly in the precision of dissection and preservation of critical anatomical structures. The enhanced dexterity and motion scaling provided by robotic systems facilitate meticulous nerve-sparing procedures and vascular control, which are crucial for postoperative functional outcomes. Additionally, robotic platforms are associated with a reduced likelihood of conversion to open surgery due to superior visualization and instrument articulation. As demonstrated in the reviewed studies, robot-assisted approaches achieve comparable, if not superior, oncological and functional outcomes, including continence recovery and complication rates, further supporting the clinical utility of robotic systems in the surgical management of prostate cancer [37].

Limitations of this study include its retrospective design, which limits causal inference between variables. Selection bias may be present due to patient recruitment from a single institution, potentially affecting the generalizability of findings to broader populations. Differences in surgical access and criteria for selecting specific surgical approaches may further contribute to this bias. Moreover, the relatively small sample size may reduce statistical power and limit the generalizability of results. Functional outcomes were assessed via telephone surveys, which may have introduced recall bias and reduced the reliability of the data. Notably, there was a disparity in surgeon experience: the laparoscopic procedures were performed by a surgeon with several hundred prior surgeries, whereas the robotic procedures were performed by a surgeon with only a few dozen completed cases at the study’s outset.

Further research is warranted to comprehensively investigate long-term clinical outcomes, assess the resumption of daily activities, and evaluate the timeframe and extent of patients’ return to occupational responsibilities following surgery. A particularly valuable area for further exploration is the detailed analysis of cost-effectiveness and economic efficiency, comparing the financial burden associated with robot-assisted and laparoscopic surgical approaches. Such an analysis should consider multiple key factors, including the duration of hospitalization, the incidence of postoperative complications, and the necessity for secondary surgical interventions. Additionally, an essential aspect to consider is the influence of the operating surgeon’s level of expertise and procedural experience on the overall surgical outcomes. A comparative evaluation of the impact of different surgical access routes on perioperative and postoperative results could also provide meaningful insights into optimizing patient management. Furthermore, an in-depth comparison of the latest generations of robotic surgical systems may yield valuable information regarding technological advancements and their potential to further refine and enhance the precision, efficiency, and safety of minimally invasive radical prostatectomy. The study, which provides compelling evidence of the superiority of the robotic method over the laparoscopic technique in selected parameters, highlights the ongoing need for the further development and refinement of robotic technology. These findings suggest that advancements in robotic-assisted surgical systems may offer substantial potential for enhancing surgical precision, improving clinical outcomes, and ultimately contributing to the betterment of the quality of life for patients undergoing radical prostatectomy.

## 5. Conclusions

The study confirmed the advantage of the robotic method over the laparoscopic method. The duration of the procedure was significantly shorter in robotic surgery than in the laparoscopic method. Furthermore, fewer complications were observed in postoperative wound healing. After hospital discharge and Foley catheter removal, urinary incontinence and UTIs were less common. Patients who underwent robotic surgery demonstrated significantly lower IPSS, compared with those who underwent laparoscopic surgery, indicating a higher quality of life. The findings of this study will be confirmed in future studies with larger patient groups.

## Figures and Tables

**Table 1:** General characteristics of the enrolled patients.

Parameter	Laparoscopic Method	Robotic Method	*p*-Value	95% CI	Passed Normality?	Used Test
Age [years], median [IQR]	70 [54–86]	69 [53–80]	0.0981	−3.464 to 0.2969	No	Unpaired *t*-test
Hospitalization duration [days], median [IQR]	5 [4–14]	5 [3–9]	0.3637	−0.8986 to 0.3319	No	Unpaired *t*-test
Surgery duration [min], median [IQR]	203.5 [86–278]	150.5 [84–172]	<0.0001	−54.00 to −28.10	No	Unpaired *t*-test
Wound complications [Y/N], n (%)	8/52 (13.3)	2/58 (3.3)	0.0946	Not relevant	Not relevant	Fisher’s exact test
Recovery room [days], median [IQR]	1 [1–3]	1 [1–4]	0.8155	−0.1578 to 0.1245	No	Unpaired *t*-test
Re-surgery [Y/N], n (%)	2/58 (3.3)	1/59 (1.7)	>0.9999	Not relevant	Not relevant	Fisher’s exact test
Transfusion of red blood cell units [Y/N], n (%)	1/59 (1.7)	1/59 (1.7)	>0.9999	Not relevant	Not relevant	Fisher’s exact test
Complications [Y/N], n (%)	12/48 (20)	10/50 (16.7)	0.8140	Not relevant	Not relevant	Fisher’s exact test
IPSS [au], mean/median	15.48/15	7.65/7	<0.0001	−10.30 to −5.377	Yes	Unpaired *t*-test
IIEF-5 [au], mean/median	4.95/0	3.21/0	0.4828	−6.711 to 3.232	No	Unpaired *t*-test
Catheterization duration [days], median [IQR]	7 [0–183]	7 [0–30]	0.1521	−21.74 to 3.456	No	Unpaired *t*-test
UTI [Y/N], n (%)	6/27 (18.2)	1/33 (2.9)	0.0544	Not relevant	Not relevant	Fisher’s exact test
Urinary incontinence [Y/N], n (%)	21/12 (63.3)	9/25 (26.5)	0.0032	Not relevant	Not relevant	Fisher’s exact test

A *p*-value < 0.05 was considered statistically significant. A *p*-value < 0.1 was considered on the borderline of significance. Abbreviations: IIEF-5, International Index of Erectile Function-5; IPSS, International Prostate Symptom Score; UTI, urinary tract infection; IQR, interquartile range.

**Table 2:** Correlations between the analyzed parameters in the laparoscopic group.

Parameter A	Parameter B	R Squared	*p*-Value
Age	Surgery duration	0.242	0.062
IIEF-5	IPSS	–0.839	<0.0001
UTI	Catheterization duration	0.356	0.042
UTI	IPSS	0.438	0.011

Abbreviations: IIEF-5, International Index of Erectile Function-5; IPSS, International Prostate Symptom Score; UTI, urinary tract infection.

**Table 3:** Correlations between the analyzed parameters in the robotic group.

Parameter A	Parameter B	R Squared	*p*-Value
Hospitalization Time	IPSS	0.307	0.078

Abbreviation: IPSS—International Prostate Symptom Score.

## Data Availability

The data used in this study are available upon reasonable request from the authors.

## Abbreviations

The following abbreviations are used in this manuscript:

LRP	Laparoscopic radical prostatectomy
RARP	Robot-assisted radical prostatectomy
UTI	Urinary tract infection
IPSS	International Prostate Symptom Score Questionnaire
IIEF-5	International Index of Erectile Function
